# Plant Proteomic Research 3.0: Challenges and Perspectives

**DOI:** 10.3390/ijms22020766

**Published:** 2021-01-14

**Authors:** Setsuko Komatsu, Jesus V. Jorrin-Novo

**Affiliations:** 1 Faculty of Environmental and Information Sciences, Fukui University of Technology, Fukui Prefecture, Fukui 910-0028, Japan; 2 Department of Biochemistry and Molecular Biology, ETSIAM, University of Cordoba, UCO-CeiA3, 14014 Cordoba, Spain

Advancements in high-throughput “Omics” techniques have revolutionized plant molecular biology research. Among them, proteomics offers one of the best options for the functional analysis of the genome, and for generating detailed information, which when integrated with that obtained by other classic and “Omics” approaches, will provide a deeper knowledge of the different plant processes. In this regard, responses to stress are key issues in plant biology research. Plants, being sessile in nature, are constantly exposed to environmental challenges resulting in substantial yield loss. To cope with harsh environments, plants have developed a wide range of adaptation strategies involving morpho-anatomical, physiological, and biochemical traits [1], knowledge of which will open new possibilities for crop breeding, improvements, and yield increase. Thus, in recent years, there has been a lot of progress in the understanding of plant responses to environmental cues at the protein level [2].

The generations of proteomics platforms (gel, label, gel free/label free, targeted) which have appeared in the last twenty years are being exploited in describing protein profiles, post-translational modifications and interactions. Nevertheless, the ultimate success of any proteomic strategy lies in various factors including the isolation, separation, visualization, and accurate identification of proteins. Despite recent advancements, more emphasis needs to be given to the protein extraction protocols, especially for very low-abundant, hydrophobic, and large molecular mass proteins. Thus, this amalgamation of diverse mass spectrometry techniques, which when complemented with genome-sequence data and modern bioinformatics analysis with improved sample preparation and fractionation strategies, offers a powerful tool to characterize novel proteins and to follow temporal changes in protein relative abundances under different environmental conditions. Furthermore, post-translational modifications and protein-protein interactions provide deeper insight into protein molecular function.

In this direction, the present “Plant Proteomics 3.0” Special Issue was conceived in an attempt to address recent advancements, as well as the limitations of current proteomic techniques and their diverse applications, to achieve new insights into plant molecular responses to various biotic and abiotic stressors and the molecular bases of other processes. Proteomic focus is also related to translational purposes, including food traceability and allergen detection. In addition, bioinformatic techniques are needed for more confident identification, quantitation, data analysis and networking, especially with non-model, orphan, plants, including medicinal and meditational plants as well as forest tree species. This Issue is the youngest brother of the previous “Plant Proteomic Research” [1] and “Plant Proteomic Research 2.0” [2] Issues. It contains 23 articles, including 4 reviews and 19 original articles. They were contributed by research groups from China, Japan, the USA, Mexico, and various European countries including Spain, Italy, Germany, the Slovak Republic, and Norway.

Four reviews deal with methodological aspects [3,4], mechanisms of ubiquitination [5], and the effect of and responses to nanoparticles treatments [6]. Smolikova et al. [3] summarized the main methodological approaches already employed in seed proteomics, as well as those still waiting for implementation in plant research, such as sample preparation, data acquisition, processing, and post-processing. Tartaglia et al. [4] focused on several soil protein extraction protocols to highlight the methodological challenges for the application of proteomics to soil samples, which enhance the identification of proteins with low abundance or from non-dominant populations. He et al. [5] summarized the latest advances in protein ubiquitination to gain comprehensive and updated knowledge, because ubiquitylation has been widely reported to be involved in many aspects of plant growth and development. Furthermore, Hossain et al. [6] highlighted the current understanding of plant responses to nanoparticles; for this purpose, the synthesis of nanoparticles, their morphophysiological effects on crops, and applications of proteomic techniques are discussed to comprehend the underlying mechanism of nanoparticles stress acclimation.

Shotgun analysis (gel-free proteomics) has supposed classical gel-based analysis (gel-based proteomics) becoming the dominant proteomic platform. One reason is that shotgun analysis affords deeper proteome coverage and allows several thousand proteins to be identified as compared to a few hundred with the gel-based approach. Both gel-based and gel-free are complementary, and the former still a valid approach [7,8,9,10]. As evidence of this, out of the 19 original papers, 15 articles report on the use of a gel-free technique. Progress has been fueled by the advancement in mass spectrometry techniques, complemented with genome-sequence data and modern bioinformatic analysis. By using two-dimensional gel electrophoresis coupled with matrix-assisted laser desorption ionization/time of flight, Sghaier-Hammami et al. [7] analyzed changes in the protein profile of non-orthodox *Quercus ilex* seeds during the maturation and germination stages, revealing some differences between orthodox/recalcitrance and viable/non-viable seeds. Lakhneko et al. [8] performed a proteomics study of grains from modern and traditional bread wheat cultivars. They used a detergent-containing buffer, which allowed the extraction of various groups of storage proteins and analyzed them by two-dimensional gel electrophoresis. Yu et al. [9] characterized glycosylated proteins in rubber latex using Pro-Q glycoprotein gel staining. Hofmann et al. [10] determined the alterations in soluble and membrane-bound class III peroxidases of maize under hypoxia by using combined proteomics and transcriptomics approaches.

Proteomics is more integrated with other “Omics” and classic approaches in the systems biology direction. Four original articles [10,11,12,13] employed proteomic and transcriptomic analyses. Hulatt et al. [11] analyzedcorrelation between proteomic and transcriptomic data, the regulation of sub-cellular localized proteins in different compartments, gene/protein functional groups, and metabolic pathways in *Nannochloropsis gaditana*. Lai et al. [12] reported that 96 genes were differentially expressed at both transcriptomic and proteomic levels in salt-tolerant and salt-sensitive hulless barley. Li et al. [13] studied at the mRNA and protein levels differences between vertical and floating leaves in sacred lotus, showing that the first is enriched in cell wall and lignin biosynthesis gene products.

In situ and single cell analysis remain some of the main challenges in proteomics studies. In this direction, Chen et al. [14] used shotgun combined with laser-capture microdissection to analyze the black layer of maize kernels, a tissue rich in stress-responsive proteins. Lawrence et al. [15] prepared stomatal guard cells from *Arabidopsis thaliana* using a Scotch-tape method to analyze changes in S-nitrosylation in guard cells during a pathogen challenge. Ceballos-Laita et al. [16] assessed the effects of the exposure of tomato roots to excess Mn on the protein profile of the xylem sap using a shotgun proteomic approach. Furthermore, plant materials are also important for the identification of unique proteins. For example, Kalunke et al. [17] reported characterization of three transgenic lines obtained from the bread wheat cultivar Bobwhite silenced by RNAi in the three ATI genes *CM3*, *CM16* and *0.28*; and these lines show unintended differences in the accumulation of high molecular mass glutenin subunits, which are involved in technological performances, but which do not show differences in terms of yield. Nawaz et al. [18] reported that semi-dwarf rice lines lacking any residual transgene-DNA and off-target effects were generated through CRISPR/Cas9-guided mutagenesis of the *OsGA20ox2* gene in a high yielding Basmati rice line, and the identified proteins were mainly enriched in the carbon metabolism and fixation, glycolysis/gluconeogenesis, photosynthesis, and oxidative phosphorylation pathways.

Proteomic techniques are used in the identification of stress-responsive mechanism in plants under conditions such as salt [14], cold [19], flood [20], and wounding [21]. Wang et al. [19] provided insights into the cold stress responses of early imbibed castor seeds: proteins confer cold tolerance by promoting protein synthesis, protect the cell against damage caused by cold stress, and facilitate resistance or adaptation to cold stress by the increased content of unsaturated fatty acid. Hashimoto et al. [20] indicated that the combined mixture of silver nanoparticles, nicotinic acid, and KNO_3_ causes positive effects on soybean seedlings by regulating the protein quality control for the mis-folded proteins in the endoplasmic reticulum, suggesting that it might improve the growth of soybeans under flooding stress. Luo et al. [21] suggested that PpAOS gene, which is involved in 12-Oxo-phytodienoic acid biosynthesis, expression enhances photosynthesis and effective energy utilization in response to wounding in *Physcomitrella patens.*

Olivares-García et al. [22] suggested that in the embryogenic culture of avocados, there is an enhanced phenylpropanoid metabolism for the production of the building blocks of lignin having a role in cell wall reinforcement for tolerating stress response. Qiu et al. [23] indicated that embryo abortion might lead to phytohormone synthesis disorder, which effected signal transduction pathways, and hereby controlled genes involved in cell wall degradation and then caused the abscission of fruitlets in sweet cherry trees. Wu et al. [24] revealed by using iTRAQ that plastid pigment metabolism contributes to leaf color changes in *Nicotiana tabacum* during curing. Xiang et al. [25] hypothesized that RPM1, a disease resistance protein, may confer resistance to *M. graminicola* in rice, and that it may possess broad-spectrum resistance against pathogens including the parasitic nematode *M. graminicola*.

Proteomics is an alive and very active discipline, and it is just one more step on the way to decipher the intricate secrets and complexity of plant systems. The guest editors hope that this special issue will provide readers with a framework for understanding plant proteomics and insights into new research directions within this field. For sure, new methodologies, approaches, equipment, and applications will be continuously appearing, and these will be the subject of “Plant Proteomic Research 4.0”. Finally, the guest editors thank all of the authors for their contributions and the reviewers for their critical assessments of these articles. Moreover, they also thank the Assistant Editor, Ms. Chaya Zeng, for giving us the opportunity to serve as guest editors for “Plant Proteomic Research 3.0”.
